# Krebserkrankungen bei Menschen mit einer Intelligenzminderung in Deutschland: Prävalenzen, Genetik und Versorgungslage

**DOI:** 10.1007/s00103-024-03837-1

**Published:** 2024-02-09

**Authors:** Tanja Sappok, Christoph Kowalski, Martin Zenker, Florian Weißinger, Andreas W. Berger

**Affiliations:** 1 Medizinische Fakultät und Universitätsklinik OWL, Krankenhaus Mara, Universitätsklinik für Inklusive Medizin, Universität Bielefeld, Maraweg 21, 33617 Bielefeld, Deutschland; 2 Deutsche Krebsgesellschaft e. V., Berlin, Deutschland; 3 Medizinische Fakultät, Universitätsklinikum Magdeburg, Institut für Humangenetik, Otto-von-Guericke-Universität Magdeburg, Magdeburg, Deutschland; 4 Klinik für Innere Medizin, Hämatologie, Onkologie, Stammzelltransplantation, Palliativmedizin, Evangelisches Klinikum Bethel, Bielefeld, Deutschland; 5 Klinik für Innere Medizin II – Gastroenterologie und gastrointestinale Onkologie, Evangelisches Krankenhaus Königin Elisabeth Herzberge gGmbH, Berlin, Deutschland; 6 Medizinisches Zentrum für Erwachsene mit Behinderungen, Evangelisches Krankenhaus Königin Elisabeth Herzberge gGmbH, Berlin, Deutschland; 7 Universitätsklinikum Ulm, Department für Innere Medizin, Klinik für Innere Medizin I, Universität Ulm, Ulm, Deutschland

**Keywords:** Onkologie, Störungen der Intelligenzentwicklung, Genetische Syndrome, Vorsorgeuntersuchungen, Mortalität, Behandlungsbesonderheiten, Oncology, Disorders of intellectual development, Genetic syndromes, Screening, Mortality, Treatment and support

## Abstract

Bei etwa 1 % der Bevölkerung besteht eine Intelligenzminderung; bezogen auf Deutschland sind das ca. 0,5–1 Mio. Menschen. Bei diesem Personenkreis ist die Lebenserwartung reduziert, wobei Krebserkrankungen zu den häufigsten Todesursachen (ca. 20 %) zählen. Trotz limitierter Datenlage kann festgestellt werden, dass das Krebsrisiko und das Sterblichkeitsrisiko im Vergleich zur Allgemeinbevölkerung erhöht sind.

Bestimmte genetische Syndrome prädisponieren für Krebserkrankungen in dieser vulnerablen Bevölkerungsgruppe, aber auch behinderungsassoziierte Komorbiditäten oder die Lebensweise könnten Risikofaktoren für onkologische Erkrankungen darstellen. Menschen mit kognitiven Beeinträchtigungen nehmen Vorsorgeuntersuchungen seltener wahr und bei der medizinischen Versorgung treten aufgrund körperlicher, kommunikativer und interaktioneller Besonderheiten Herausforderungen auf. Eine optimierte Zusammenarbeit von spezialisierten Fachkliniken für Menschen mit Behinderungen und den jeweiligen Krebszentren ist erforderlich, um die Prozesse individuell auf die krebskranke Person zuzuschneiden.

In Deutschland fehlen Daten zu den Prävalenzen einzelner Tumorentitäten, der Inanspruchnahme und des Bedarfs von Gesundheitsleistungen. Es ist dringend notwendig, den Themen Krebsprävention, -therapie und -forschung in der vulnerablen und heterogenen Patientengruppe von krebserkrankten Menschen mit einer Intelligenzminderung Aufmerksamkeit zu widmen, um dem Anstieg der krebsbedingten Todesfälle in dieser Bevölkerungsgruppe wirksam zu begegnen.

Der Beitrag fasst Fachwissen zu Krebserkrankungen bei Menschen mit einer kognitiven Beeinträchtigung zusammen, arbeitet Besonderheiten in der Behandlung heraus, stellt Versorgungsstrukturen dar und leitet konkrete Bedarfe für Klinik und Forschung ab.

## Abstract (en)

Intellectual disability has a prevalence rate of approximately 1% of the population; in Germany, this is around 0.5–1 million people. The life expectancy of this group of people is reduced, with cancer being one of the most common causes of death (approx. 20%). Overall, the risk of cancer and mortality is increased compared to the general population.

Certain genetic syndromes predispose to cancer in this vulnerable group, but associated comorbidities or lifestyle could also be risk factors for cancer. People with cognitive impairments are less likely to attend preventive check-ups, and challenges arise in medical care due to physical, communicative, and interactional characteristics. Optimized cooperation between clinical centers for people with disabilities and the respective cancer centers is required in order to tailor the processes to the individual.

In Germany, there is a lack of data on the prevalence of cancer entities and the use and need for healthcare services. There is an urgent need to focus attention on cancer prevention, treatment, and research in the vulnerable and heterogeneous group of people with intellectual disabilities suffering from cancer in order to effectively counteract the increase in cancer-related deaths in this population group.

The article summarizes specialist knowledge on cancer in people with an intellectual disability, identifies special features of treatment, presents care structures, and derives specific requirements for clinics and research.

## Einleitung

Auch wenn Krebserkrankungen bei Menschen mit kognitiven Beeinträchtigungen (Intelligenzminderung, Störung der Intelligenzentwicklung) wie bei Menschen ohne Behinderungen auftreten können, ist das Fachwissen zu den spezifischen Bedarfen im Hinblick auf Prävention, Diagnostik und Behandlung bei diesem Personenkreis begrenzt. Durch die insgesamt gestiegene Lebenserwartung – bei Menschen mit wie ohne Behinderungen – ist das Gesundheitswesen vermehrt auch mit an Krebs erkrankten Menschen mit einer kognitiven Beeinträchtigung konfrontiert und aufgefordert, eine optimale Behandlung zu gewährleisten. Neben einem barrierefreien Zugang zu präventiven Untersuchungen sind die ambulante und stationäre Abklärung und Therapie sicherzustellen.

Unter einer Intelligenzminderung (ICD[International Classification of Diseases]-10: F7x) bzw. Störung der Intelligenzentwicklung (ICD-11: 6A00) versteht man deutlich unterdurchschnittliche intellektuelle Leistungen (2 Standardabweichungen unter Mittelwert; < 2,3. Perzentil) und reduzierte Adaptationsfähigkeit in den Bereichen *Konzeptualisierungsfähigkeit, soziale Fähigkeiten* und *alltagspraktische Fähigkeiten*, die während der Entwicklungsperiode entstehen. Artikel 25 der Behindertenrechtskonvention der Vereinten Nationen (UN) zur gesundheitlichen Teilhabe sieht vor, dass Menschen mit Behinderungen ein Recht „auf das erreichbare Höchstmaß an Gesundheit ohne Diskriminierung aufgrund von Behinderung“ haben.^1^ Diese Vorgabe schließt auch onkologische Erkrankungen ein.

Zur Beurteilung des Umsetzungsgrads der Konvention wären Versorgungs- und Bedarfsanalysen wichtig. In Deutschland fehlen Daten zu den Prävalenzen einzelner Tumorentitäten, der Inanspruchnahme und des Bedarfs von Gesundheitsleistungen. Krebsdiagnosen mitsamt der Klassifikation werden hierzulande nicht systematisch dokumentiert. Dieses Problem setzt sich bei der Beschreibung von Inanspruchnahme und Bedarfen einer spezifischen gesundheitlichen Diagnostik und Therapie fort [1].

Ziel des Übersichtsartikels ist es, Fachwissen u. a. zu genetischen Faktoren bei Krebserkrankungen bei Menschen mit einer kognitiven Beeinträchtigung zusammenzufassen, Besonderheiten in der Behandlung herauszuarbeiten, die Versorgungsstrukturen darzustellen und konkrete Bedarfe für Klinik und Forschung abzuleiten.

## Fallbeispiel

Im Mai 2017 entwickelte eine damals 45-jährige Patientin mit Down-Syndrom schleichende Symptome wie Gangunsicherheit und Schwäche der linken Körperhälfte. Durch die langsame Symptomentwicklung und die Zurückhaltung der Patientin gegenüber ärztlichen Maßnahmen war die Abklärung etwas verzögert.

Nach der Bildgebung des Kopfes mit CT und MRT wurde der Verdacht auf eine entzündliche Veränderung oder ein Lymphom gestellt. Die Diagnostik konnte bei der Patientin mit sehr guter Unterstützung durch ihre Schwester, die zeitgleich rechtliche Betreuerin war, durchgeführt werden. Die cMRT-Untersuchung und die Liquorpunktion waren nur in Vollnarkose möglich.

In der Tumorkonferenz wurde empfohlen, den Befund – in Narkose – zu biopsieren. Mittels osteoplastischer Trepanation wurde im Juli 2017 ein aggressiver Tumor im Sinne eines primär zerebralen Lymphoms diagnostiziert. Da die Patientin ansonsten gesund war, wurde in der Tumorkonferenz eine Zentralnervensystem(ZNS)-gängige, intensive Chemotherapie empfohlen. Die wirksamsten Substanzen für diese Erkrankung sind hochdosiertes Methotrexat, hochdosiertes Cytarabin, Thiotepa und Rituximab. Während die diagnostischen Maßnahmen im Rahmen der Einschätzung des Ausmaßes einer Krebserkrankung (Staging) noch zum Teil in Sedierung bzw. Vollnarkose durchgeführt werden konnten, war die Chemotherapie auf verschiedenen Ebenen herausfordernd:

- die Akzeptanz von langdauernden Infusionsbehandlungen,
- die Akzeptanz von teils mehrwöchigen stationären Aufenthalten,
- das Tolerieren von Nebenwirkungen, wie Schleimhautschädigungen, Übelkeit und Appetitlosigkeit,
- die Akzeptanz von Hygienemaßnahmen in Phasen der ausgeprägten Abwehrschwäche.

Dies über mehrere Zyklen durchzuhalten, forderte die Patientin, ihre Schwester und das Behandlungsteam heraus. Die Schwester der Patientin wurde gebeten, diese bei längeren Infusionen zu begleiten und zu unterstützen, wodurch diese die Behandlung besser akzeptieren konnte. Der Patientin war es wichtig, nur von vertrauten Personen des Teams betreut und mit Vornamen angesprochen zu werden. Die Kunsttherapie war für die Patientin eine große Stütze, da sie sich mit Malen in den langen Aufenthalten auf der Station ablenken konnte.

Konstante Bezugspersonen in der Pflege, das Therapeuten- und Ärzteteam halfen der Patientin, die Behandlung anzunehmen und die wiederholten Krankenhausaufenthalte durchzustehen. Durch ausführliche und zum Teil spielerische Erklärungen in leichter Sprache konnte sie die notwendigen Hygienemaßnahmen verstehen und akzeptieren.

Die Patienten wurde vor den Therapiezyklen über mögliche Nebenwirkungen informiert, sodass sie davon nicht überrascht wurde und Vertrauen in die Behandlung aufbaute. Der zeitliche Verlauf zur weiteren Entwicklung der Nebenwirklungen war schwierig zu vermitteln, wobei das Arbeiten mit konkreten für die Patientin relevanten Orientierungspunkten (bestimmte Feiertage, Wochenenden) hilfreich war.

Weiterhin wurde die Chemotherapie angepasst, um sie für die Patientin machbar zu gestalten. Es wurde auf die Gabe von hochdosiertem Methotrexat verzichtet, die mit einem mehrtägigen Infusionsprogramm eine große Belastung dargestellt hätte und mit einem hohen Risiko für schwere Nieren‑, Leber- oder ausgeprägter Hautschädigung verbunden ist.

Nach jedem Chemotherapiezyklus wurde die Zeit der Neutropenie mit hohem Infektionsrisiko durch die Gabe von Granulozyten-Kolonie-stimulierendem Faktor (G-CSF) reduziert. So wurde die Zeit der Therapie ab Juli 2017 mit 5 Zyklen Immun-Chemotherapie (Rituximab, hochdosiertes Cytarabin und Thiotepa) über 4 Monate für die Patientin durchhaltbar gestaltet.

Erfreulicherweise konnte eine komplette Remission erreicht werden, die bis August 2018 bildgebend kontrolliert wurde. Im weiteren Verlauf wurde auf die cMRT-Untersuchung verzichtet, da sie nur in Vollnarkose durchgeführt werden konnte.

Bei der Behandlungsplanung und -durchführung war zentral, immer wieder die konkrete Machbarkeit und Umsetzbarkeit für die Patientin einerseits und den zu erwartenden Nutzen durch die Behandlung andererseits gegeneinander abzuwägen. Hier gibt es keine generell gültigen Orientierungswerte, dies muss ganz individuell auf der Basis der jeweiligen Fähigkeiten, Bedürfnisse, sozialen Netzwerke und Krankheitsbilder im Sinne einer „informierten Entscheidungsfindung“ mit den Betroffenen und ihren Angehörigen bzw. rechtlich Betreuenden erfolgen.

## Intellektuelle Beeinträchtigung und Krebs

Trotz der noch immer insgesamt reduzierten Lebenserwartung erleben wir gegenwärtig die erste Generation alt werdender Menschen mit kognitiver Beeinträchtigung in Deutschland. Damit steigt auch die Wahrscheinlichkeit für die Entwicklung altersassoziierter Krankheitsbilder wie Krebserkrankungen. Die häufigsten Todesursachen bei diesem Personenkreis sind Kreislauf- und Atemwegserkrankungen [2], gefolgt von Malignomen, die ca. 15–20 % der Todesfälle ausmachen [3–5].

Interessanterweise waren in einer populationsbasierten Studie Neoplasien bei Menschen mit kognitiver Beeinträchtigung zwar für 14,9 % der Todesfälle ursächlich im Vergleich zu 37,4 % in der Vergleichspopulation. Die Hazard Ratio (HR) für Tod an einer Krebserkrankung war dennoch bei Menschen mit kognitiver Beeinträchtigung mit 1,44 signifikant erhöht [3]. Diese Unterschiede lassen sich möglicherweise dadurch erklären, dass einige maligne Erkrankungen in dieser Patientenpopulation unterdiagnostiziert oder nicht ausreichend (zum Beispiel an Krebsregister) gemeldet werden [6]. Dennoch verdeutlichen diese Daten, dass u. U. unterschiedliche zugrunde liegende biologische Funktionszustände relevanter sind, was den insgesamt größeren Gesundheitsbedarf von Menschen mit kognitiver Beeinträchtigung verdeutlicht. Zu ähnlichen Ergebnissen einer etwa 1,5-fach erhöhten Mortalität durch Krebserkrankungen bei Menschen mit kognitiver Beeinträchtigung kam eine Studie aus den Niederlanden [7].

Grundsätzliche kognitive Beeinträchtigungen als mitverursachende, aber nicht zugrunde liegende Ursachen sollten daher in Sterbeurkunden besser registriert werden, um reale Morbiditäts- und Mortalitätsanalysen durchführen zu können und dadurch die Gesundheitsversorgung dieser Menschen in allen Ländern zu verbessern [8].

Das Wissen über Krebserkrankungen bei dem genannten Personenkreis ist noch gering. Klinisch Tätige beschreiben den Eindruck, dass Menschen mit kognitiven Beeinträchtigungen Vorsorgeuntersuchungen seltener wahrnehmen, später diagnostiziert werden und oft nicht dieselbe Bandbreite an Therapieoptionen angeboten bekommen wie die Allgemeinbevölkerung [1].

Möglicherweise haben genetische Faktoren, behinderungsassoziierte Komorbiditäten oder die Lebensweise von Menschen mit Behinderungen einen Einfluss auf das Spektrum der zu beobachtenden Krebserkrankungen. Dies wurde von O’Leary et al. (2018) diskutiert, die in ihrem systematischen Review von Mortalitätsstudien bei Menschen mit einer Intelligenzminderung feststellten, dass der Anteil gastrointestinaler Krebserkrankungen an den krebsbedingten Todesfällen im Vergleich zur Allgemeinbevölkerung besonders hoch sei [9]. Detaillierte Untersuchungsergebnisse und weitere Forschung dazu fehlen.

Vielversprechend ist insbesondere der Blick auf durch Früherkennung vermeidbare Erkrankungen und Todesfälle. Ein europäisches Forschungsprojekt, die COST-Aktion CA21123 „Cancer–Understanding Prevention in Intellectual Disabilities“, ist ein Forschungsverbund zur Prävention von Krebserkrankungen bei Menschen mit Behinderungen.^2^ Ziel ist, europaweit Krebspräventionsstrategien für Menschen mit einer Intelligenzminderung zu identifizieren und zu verbessern.

## Fakten zu Gesundheit und Krankheit

Menschen mit einer in der Entwicklungsperiode entstandenen kognitiven Beeinträchtigung machen ungefähr 1 % der Bevölkerung in den westlichen Ländern aus [10]. Etwa 2 Drittel dieser Behinderungsformen sind genetisch bedingt, wobei insbesondere bei schweren Behinderungsformen häufig Neumutationen beobachtet werden [11]. In Deutschland leben ca. 0,5–1 Mio. Menschen mit einer Intelligenzminderung und diese Personengruppe hat ein erhöhtes Risiko für körperliche und psychische Erkrankungen [12].

Trotz der hohen Krankheitslast gibt es viele Hindernisse und Herausforderungen, um eine angemessene Gesundheitsversorgung zu ermöglichen (vgl. Fallbeispiel). Dabei spielen sowohl patientenbezogene Faktoren eine Rolle, z. B. bzgl. Gesundheitskompetenz und Kommunikation, als auch aufseiten der Behandelnden bestehende Barrieren wie unzureichendes Wissen oder gar diskriminierende Einstellungen [13].

Gesundheitliche Beeinträchtigungen führen zu einer erheblichen emotionalen, sozialen und finanziellen Belastung der Personen selbst und ihrer sozialen Netzwerke. Dies gilt auch für onkologische Erkrankungen. Über Krebserkrankungen bei Personen mit kognitiver Beeinträchtigung in Deutschland gibt es bisher nur begrenztes Wissen.

## Prävalenz von Krebserkrankungen bei Menschen mit kognitiver Beeinträchtigung

In Deutschland werden jedes Jahr rund 500.000 neue Krebsdiagnosen gestellt [14]. Zur Inzidenz von Krebserkrankungen bei Menschen mit kognitiver Beeinträchtigung gibt es dagegen aus Deutschland keine Zahlen und weltweit nur wenige aussagekräftige epidemiologische Studien.

Eine auf nationalen Registern beruhende Studie aus Schweden schloss über 3,5 Mio. Personen ein, davon 28.000 Menschen mit kognitiver Beeinträchtigung der Geburtsjahrgänge 1974–2013 (Altersspektrum Kindheit bis 43 Jahre). Diese Studie ermittelte ein erhöhtes relatives Risiko (HR 1,57) für onkologische Erkrankungen bei Menschen mit einer kognitiven Beeinträchtigung [15]. Dieselbe Arbeitsgruppe untersuchte Krebsdiagnosen bei Menschen mit Autismus-Spektrum-Erkrankungen (Beobachtungszeit bis 30 Jahre) und beschreibt eine höhere Krebsinzidenz bei diesem Personenkreis, wobei hierbei ggf. komorbide Intelligenzminderungen oder Geburtskomplikationen vorlagen (Odds Ratio [OR] 1,3); werden die komorbiden Störungen rausgerechnet, liegt die OR für Menschen mit einer Autismus-Spektrum-Störung bei 1,0.

Studien, die ältere Erwachsene einschlossen, aber mit deutlich geringeren Größen der untersuchten Kohorten und geringerer statistischer Teststärke, zeigten keine generelle Risikoerhöhung bei Menschen mit kognitiver Beeinträchtigung [16, 17].

Die Risikoverteilungen zwischen den Geschlechtern und spezifische Risiken für einzelne Krebsentitäten ergeben in den verschiedenen Studien insgesamt kein einheitliches Bild. Mehrere Studien ermittelten bei Menschen mit kognitiver Beeinträchtigung besonders erhöhte Risiken für Tumoren des Gastrointestinaltraktes, vor allem für das Ösophaguskarzinom [18]. Das Krebserkrankungsrisiko kann auch bei bestimmten Syndromen deutlich gesteigert sein [19]. Aus Krebsregistern lassen sich für Deutschland keine Aussagen treffen, da Krebsdiagnosen ohne zusätzliche Beschreibung von Nebendiagnosen, wie z. B. Intelligenzminderung (ICD-10 F7x), erfasst werden. Angesichts dieser Datenlage lassen sich Inzidenz- und Mortalitätsraten schwer beschreiben. Dies ist aber nötig, um effektive Präventionsstrategien für vulnerable Gruppen zu etablieren.

Zusammenfassend kann man trotz der gegebenen Limitationen der Datenlage feststellen, dass generell bei Menschen mit kognitiver Beeinträchtigung Krebsrisiken zumindest im Kindes- bis zum mittleren Erwachsenenalter (40 Jahre) erhöht sind und dass die Mortalität an Tumorerkrankungen bei Menschen mit kognitiver Beeinträchtigung über der der Allgemeinbevölkerung liegt.

## Einfluss genetischer Faktoren

Bei der Mehrheit der Personen mit einer kognitiven Beeinträchtigung liegt eine genetische Ursache der Störung vor. Diese kann direkt Auswirkungen auf das Risiko zur Tumorentstehung haben. Tatsächlich ist heute klar, dass viele für kognitive Beeinträchtigung ursächliche Gene eine wichtige Rolle für molekulare Mechanismen und Signalwege spielen, deren Störungen auch wesentlich für die Tumorentstehung sind [20]. Etwa ein Drittel der Gene, in denen somatische tumorassoziierte Veränderungen bekannt sind, haben gleichzeitig bei Keimbahnveränderungen eine Assoziation mit kognitiver Beeinträchtigung [21].

Erkrankungsgruppen, für die das zutrifft, sind u. a. Phakomatosen (z. B. tuberöse Sklerose, Neurofibromatose), RASopathien (z. B. Noonan-Syndrom, Costello-Syndrom), Erkrankungen des PI3K-AKT-Signalwegs (z. B. Cowden-Syndrom), das große Spektrum der Störungen der Chromatinregulation (Cornelia-de-Lange-Syndrom, Kabuki-Syndrom, Coffin-Siris-Syndrom, Rubinstein-Taybi-Syndrom, Sotos-Syndrom u. v. a. m.) sowie Chromosomen-Instabilitätssyndrome (z. B. Ataxia teleangiectasia, Nijmegen-Breakage-Syndrom, LIG4-Syndrom; [20]).

Für einen relevanten Einfluss der zugrunde liegenden genetischen Veränderungen auf die erhöhten Tumorrisiken bei kognitiver Beeinträchtigung sprechen auch die o. g. Beobachtungen erhöhter Tumorrisiken bei Autismus-Spektrum-Störungen mit Intelligenzminderung und/oder angeborenen Fehlbildungen, für welche häufig genetische Veränderungen verantwortlich sind. Dies steht im Gegensatz zum hochfunktionalen Autismus, der selten durch monogenetische Veränderungen verursacht wird.

Die Bedeutung genetischer Faktoren für die erhöhten Tumorrisiken bei Menschen mit kognitiver Beeinträchtigung zeigt sich noch klarer in der Betrachtung einzelner Erkrankungen. Beispielsweise geht das Down-Syndrom mit erhöhten Risiken für akute Leukämien (myeloisch und lymphatisch) sowie für Hodenkrebs einher. Das höchste Risiko für Leukämien wurde bei Kindern und jungen Erwachsenen gesehen, ab dem Alter von 30 Jahren wurde kein erhöhtes Leukämierisiko mehr beobachtet [22].

Zu den häufigeren genetisch bedingten kognitiven Beeinträchtigungen mit gut belegten, moderat erhöhten Tumorrisiken gehören neben dem Down-Syndrom auch die tuberöse Sklerose; bei diesem Syndrom sind die wichtigsten Tumorentitäten benigne und selten maligne Angiomyolipome der Nieren (70 % bzw. 1 %), Nierenzellkarzinom (2–5 %) und subenpendymale Riesenzellastrozytome des Gehirns (5–15 %; [23]). Bei der Neurofibromatose Typ 1, die mit einer milden kognitiven Beeinträchtigung assoziiert sein kann, finden sich neben den obligatorischen kutanen Neurofibromen unter anderem Optikusgliome (15–20 %, überwiegend im Kindesalter), maligne Nervenscheidentumoren (8–13 %), Brustkrebs (ca. 20 %), Phäochromozytome und gastrointestinale Stromatumoren [24]. Manche Formen der kognitiven Beeinträchtigung können mit sonst sehr ungewöhnlichen Tumoren assoziiert sein, wie beispielsweise beim Rubinstein-Taybi-Syndrom, bei dem Pilomatrixome (gutartige epitheliale Neoplasien follikulären Ursprungs) deutlich gehäuft vorkommen [25].

Für viele andere Formen von kognitiver Beeinträchtigung mit monogener, aber auch chromosomaler Ursache gibt es Hinweise auf mögliche erhöhte Tumorrisiken. Diese Vermutungen beruhen aber oft auf pathophysiologischen Gemeinsamkeiten mit tumorgenetischen Mechanismen und einer begrenzten Zahl kasuistischer Beschreibungen in kleinen Kohorten. Epidemiologische Daten, die erhöhte Risiken robust belegen und genauer beziffern, sind selten verfügbar.

Weiterhin gibt es sehr seltene Formen der kognitiven Beeinträchtigung mit stark erhöhten Tumorrisiken. Dazu gehören Chromosomen-Instabilitätssyndrome wie die Ataxia teleangiectatica (mind. 25 % Risiko für Leukämien und Lymphome, bei Erwachsenen zusätzlich auch erhöhte Risiken für ein breites Spektrum solider Tumoren) und das Nijmegen-Breakage-Syndrom (deutlich erhöhtes Risiko für Lymphome und andere Tumoren von mind. 60 % bereits bis zum Alter von 25 Jahren). Bei Syndromen aus dieser Gruppe ist das Wissen um die Grunderkrankung besonders wichtig, weil im Rahmen einer Tumortherapie die erhöhte Strahlen- und Chemotherapie-Sensibilität zu berücksichtigen sind.

Sehr hohe Tumorrisiken haben auch Menschen mit kognitiver Beeinträchtigung aufgrund von seltenen chromosomalen Deletionen („contiguous gene deletions“), welche Gene für bekannte familiäre Tumorprädispositionen enthalten. Beschrieben wurden syndromale Formen von kognitiver Beeinträchtigung mit Deletionen in 13q, die das *RB1*-Gen enthalten (familiäres Retinoblastom; [26]), Deletionen in 5q, die das *APC*-Gen enthalten (familiäre Polyposis; [27]), Deletionen in 2p, die die Gene *MSH2* und *MSH6* enthalten (hereditäres nonpolypöses Kolonkarzinom; [28]). Die Betroffenen haben dann neben der kognitiven Beeinträchtigung auch die spezifischen Tumorrisiken, die zu der jeweiligen familiären Tumorprädispositionserkrankung gehören, und bedürfen entsprechender Früherkennungs- und Therapiemaßnahmen.

Auch nicht-genetische Faktoren können zu der erhöhten Krebsinzidenz bei Menschen mit kognitiver Beeinträchtigung beitragen. Risikofaktoren für onkologische Erkrankungen wie sitzende Lebensweise, verminderte körperliche Aktivität und Übergewicht kommen bei Erwachsenen mit kognitiver Beeinträchtigung häufiger vor [29]. Für die beobachtete höhere Prävalenz des Ösophaguskarzinoms bei Menschen mit kognitiver Beeinträchtigung wurde ein möglicher Zusammenhang mit einem chronischen gastroösophagealen Reflux vermutet [15].

## Herausforderungen in der medizinischen Versorgung bei Menschen mit kognitiver Beeinträchtigung

Eingeschränkte Kommunikations- und Introspektionsfähigkeiten erschweren die Anamneseerhebung. Die Beeinträchtigung selbst kann im Sinne einer diagnostischen Überschattung*, *dem sogenannten „diagnostic overshadowing“, die Diagnostik verkomplizieren, insbesondere wenn sich Krankheitsbilder und mit der Behinderung verbundene Einschränkungen überlagern und schwer voneinander differenzieren lassen [30]. Krankheitsbilder und -verläufe können ungewöhnlich sein und die Bewältigungsmöglichkeiten („coping“) sind reduziert (vgl. Fallbeispiel). Zusätzliche körperliche Erkrankungen (Spastik, Epilepsie) und sensorische Beeinträchtigungen beeinflussen den diagnostischen und therapeutischen Prozess und das Wohlbefinden. Häufige Störungsbilder, wie z. B. Autismus-Spektrum-Störungen, sind mit sensorischen, kommunikativen und interaktionellen Besonderheiten verbunden, die eine gelungene Zusammenarbeit weiter herausfordern. Diagnostische Wege und therapeutisches Handeln müssen daher individuell auf den Patienten bzw. die Patientin mit komplexer Mehrfachbehinderung zugeschnitten werden.

Gesicherte Informationen über die onkologische Versorgungsqualität bei Personen mit kognitiver Beeinträchtigung fehlen. Es mangelt an formulierten Standards und Patientenpfaden, die die Versorgungsqualität abbilden. Üblicherweise werden diese in zertifizierten Zentren über Qualitätsindikatoren, die aus den Leitlinien abgeleitet werden, erfasst [31]. Für Personen mit kognitiver Beeinträchtigung gibt es allerdings weder spezifische Empfehlungen in den onkologischen Leitlinien noch spricht die aktuelle S2k-Leitlinie „Intelligenzminderung“ Empfehlungen zur onkologischen Versorgung aus [32]. Erfahrungsberichte medizinischer Leistungserbringender legen nahe, dass Krebspatientinnen und -patienten mit kognitiver Beeinträchtigung in Deutschland eher keine gleichberechtigte oder bedarfsgerechte Versorgung erfahren [1], was sich beispielsweise in einer verspäteten Diagnose niederschlägt. Dass es an klaren Empfehlungen fehlt, liegt auch an der weitgehend fehlenden spezifischen Evidenz für Personen mit kognitiver Beeinträchtigung (vgl. Fallbespiel).

### Gelingende medizinische Versorgung

Auf der individuellen Ebene gelingen die medizinische Abklärung und Behandlung von Menschen mit einer kognitiven Beeinträchtigung in enger Kooperation mit Angehörigen, Betreuenden und den weiteren Lebensfeldern (Vereine, Arbeit etc.) der Betroffenen. Niedrigschwellige und kostengünstige Informationsangebote, wie z. B. Materialien in leichter Sprache [33], oder Methoden der unterstützten Kommunikation erleichtern den Behandlungsprozess.

In einem gemeinsamen Projekt der Bundesvereinigung Lebenshilfe und der Deutschen Krebsgesellschaft entstanden umfassende Informationsmaterialen speziell zu Krebserkrankungen („Lucy bekommt eine Krebs-Behandlung. Informationen in Leichter Sprache über die Behandlung von Krebs“) und zur Früherkennung („Monika geht zur Brustkrebs-Vorsorge, Jan geht zur Darmkrebs-Vorsorge, Sarah macht einen Abstrich. Informationen in Leichter Sprache über die Krebs-Vorsorge“).^3^

Die Versorgungsforschung als diejenige Disziplin, die sich damit befasst, was an Leistungen unter Alltagsbedingungen letztlich bei den Betroffenen ankommt [34], nimmt sich des Themas erst langsam an. Eine deutsche Studie aus dem Jahr 2017 untersuchte die generelle Zugänglichkeit von Menschen mit kognitiver Beeinträchtigung zu Gesundheitsuntersuchungen. Sie war nicht krebsspezifisch, konnte aber wichtige Erkenntnisse für die onkologische Versorgung liefern. Dabei wurden aufwändigere oder invasive Prozeduren zur Vorsorge eines Brust‑, Dickdarm- oder Prostatakarzinoms von Menschen mit kognitiver Beeinträchtigung seltener in Anspruch genommen [35].

Information und Beratung sind zwar nur ein kleiner, aber dennoch wichtiger Beitrag auf dem Weg zu einer bedarfsgerechten Versorgung. Darüber hinaus gibt es Vorschläge zur Verbesserung der Versorgungssituation, darunter die Sicherstellung des Zugangs zu Früherkennungsmaßnahmen, die Finanzierung der Assistenzbegleitung im Krankenhaus und die Fort- und Weiterbildung von Personal im Gesundheitswesen. Ein wichtiger Baustein in der Verbesserung der Versorgung von krebskranken Personen mit kognitiver Beeinträchtigung dürften zertifizierte Krebszentren [36] sein.

## Onkologische Vorsorge bei Menschen mit kognitiver Beeinträchtigung

Konkrete Früherkennungsempfehlungen sind nur für sehr wenige Erkrankungen formuliert. Umso wichtiger sind die Sensibilisierung und Aufklärung der Personen selbst, ihrer Familien und Betreuungseinrichtungen sowie die konsequente Durchführung der allgemein empfohlenen Früherkennungsuntersuchungen. Für viele seltene genetisch bedingte kognitive Beeinträchtigungen mit bekannter Tumorprädisposition sind die Lebenszeitrisiken nicht bekannt. Ob bei einer kognitiven Beeinträchtigung mit belegter Tumorprädisposition im Kindesalter diese auch im weiteren Leben mit erhöhten Tumorrisiken verbunden ist, ist meist nicht geklärt.

Selbst die Durchführung allgemein empfohlener Früherkennungsmaßnahmen wie Darmkrebsvorsorge, gynäkologische Vorsorge oder Hautkrebsscreening kann bei Menschen mit kognitiver Beeinträchtigung eine besondere Herausforderung darstellen. Mehrere Studien aus verschiedenen Ländern weisen eine deutlich reduzierte Inanspruchnahme der allgemein empfohlenen Krebsfrüherkennungsmaßnahmen am Beispiel des Brustkrebses in dieser Patientengruppe nach [37–39]. Dies passt zur Analyse ambulanter Abrechnungs- und Arzneiverordnungsdaten aus Deutschland aus dem Jahr 2018, die zeigten, dass 36 % der Menschen mit kognitiver Beeinträchtigung keine spezialisierte Facharztbehandlung in Anspruch nahmen [40].

Für Deutschland existieren zur Wahrnehmung der Früherkennungsuntersuchungen bisher keine Zahlen, aber es ist anzunehmen, dass ähnliche Barrieren wie in anderen westlichen Ländern gegeben sind. Als solche Barrieren wurden diskutiert: Angst, Stress und Scham, fehlendes Wissen, Vorbereitung und Einwilligungs- und Kommunikationsfähigkeit sowie Mobilitätseinschränkungen [41].

Zu den erhöhten allgemeinen Krebsrisiken bei Menschen mit kognitiven Beeinträchtigungen tragen neben Lebensweise und -umständen auch seltene genetischen Veränderungen mit stark erhöhtem Krebsrisiko bei (s. oben). Daher ist es einerseits wichtig, die genetische Grundlage einer kognitiven Beeinträchtigung zu kennen, und andererseits im individuellen Kontext entscheidend, den Bedarfen dieser heterogenen Patientengruppe gerecht werden zu können.

## Medizinische Versorgungsangebote für Menschen mit kognitiver Beeinträchtigung und Krebserkrankungen

Der Mangel an barrierefreien Kliniken und ambulanten Angeboten verzögert die Erkennung und Behandlung von Krebserkrankungen für Menschen mit kognitiver Beeinträchtigung [42]. Unter den gegenwärtigen Rahmenbedingungen ist in Deutschland keine barrierefreie Gesundheitsversorgung von Menschen mit Behinderungen gewährleistet [43].

Dem Bedarf hat der Gesetzgeber mit § 119c sowie § 43b (Sozialgesetzbuch V) Rechnung getragen, die einen Rechtsanspruch auf eine spezialisierte Versorgung in sogenannten Medizinischen Behandlungszentren für Erwachsene mit geistiger oder schwerer Mehrfachbehinderung (MZEB) vorsehen. Inzwischen existieren in Deutschland über 50 solcher Zentren. Mit regional unterschiedlichen fachlichen Schwerpunkten wird hier ein interdisziplinäres und multiprofessionelles Behandlungsangebot gemacht. Die Behandlung im MZEB setzt voraus, dass aufgrund der Schwere, der Art und der Komplexität der Behinderung eine besondere und individuell ausgerichtete Behandlung erforderlich ist, die nicht im Rahmen der regulären haus- oder fachärztlichen Versorgungsangebote möglich ist.

Problematisch ist, dass in vielen Fällen nur die Diagnostik und „Behandlungserprobung“, nicht aber die eigentliche Behandlung finanziert wird. Ein gelungenes Zusammenspiel aller Akteure, sowohl zwischen den verschiedenen Fachrichtungen und Berufsgruppen (Stichwort „inklusive Medizin“) als auch zwischen den Selbstvertretenden, den Einrichtungen der Eingliederungshilfe, den Angehörigen und den Städten und Gemeinden, ist nötig, um die Gesundheitsversorgung zu optimieren [44].

Gegenwärtig gibt es keine flächendeckende Versorgungsstruktur für die stationäre Behandlung von Menschen mit Behinderungen. Gerade im Kontext von Krebserkrankungen ist hier eine optimierte Zusammenarbeit von spezialisierten Fachkliniken für Menschen mit Behinderungen und den jeweiligen Krebszentren erforderlich, sowohl in der Behandlungsplanung in den Tumorkonferenzen als auch in der Behandlungsdurchführung. Vor diesem Hintergrund wurde von den Fachgesellschaften und Verbänden für Menschen mit Behinderungen ein gemeinsamer Appell verfasst.^4^

Die genannten Initiativen zielen darauf ab, auf Systemebene den barrierefreien Zugang zu präventiven, kurativen, rehabilitativen und ggf. auch palliativen Versorgungsstrukturen für Menschen mit Behinderung sicherzustellen. Dafür hat die Deutsche Gesellschaft für seelische Gesundheit bei geistiger Behinderung (DGSGB) dem Bundesgesundheitsministerium im Rahmen des Aktionsplans für ein Inklusives Gesundheitswesen einen Vorschlag zur Verbesserung der stationären Behandlung von Menschen mit Intelligenzminderung gemacht.

Das Stufenkonzept sieht eine Verbesserung erstens der stationären Regelversorgung durch Kompetenzteams, zweitens der regionalen Versorgung durch spezialisierte, stationäre Betteneinheiten und drittens in Unikliniken bzw. universitär angebundenen interdisziplinären Fachkliniken vor, wo neben der Versorgung von Schwerstkranken auch Forschung sowie Aus‑, Fort- und Weiterbildung stattfinden kann.^5^ Daneben ist ein personenzentriertes und individuell gestaltetes Vorgehen sinnvoll (s. Fallbeispiel).

## Fazit: Forschungs- und Entwicklungsbedarfe für die Zukunft

Es ist dringend notwendig, der vulnerablen Patientengruppe von Menschen mit einer Intelligenzminderung und einer Krebserkrankung dezidierte Aufmerksamkeit in puncto Krebsprävention, -therapie und -forschung zu widmen, um dem Anstieg der krebsbedingten Todesfälle in dieser Bevölkerungsgruppe wirksam zu begegnen.

Langzeitstudien an Erwachsenen und älteren Menschen sind nötig, um erkrankungsspezifische Krebsrisiken genauer zu definieren. Spezifische Maßnahmen können die Inanspruchnahme von Früherkennungsmaßnahmen weiter verbessern.

## Footnotes

1 https://www.behindertenrechtskonvention.info/gesundheitssorge-3910/ Zugriff am 10.11.2023.

2 Der Autor Andreas Berger und die Autorin Tanja Sappok sind vom Bundesministerium für Bildung und Forschung als Vertretende für Deutschland für den Forschungsverbund nominiert worden.

3 Die Auflage von 20.000 Exemplaren ist mittlerweile vergriffen, aber die Büchlein sind als PDF zum Download verfügbar: https://www.lebenshilfe.de/informieren/familie/krebsinformationen-ls, Zugriff am 10.11.2023.

4 https://www.lebenshilfe.de/fileadmin/Redaktion/PDF/Wissen/public/Gemeinsame-Stellungnahmen/A4_Stellungnahme_Papier_zur_spezalisierten_Versorgung_Gemeinsamer_Appell.pdf, Zugriff am 10.11.2023.

5 https://dgsgb.de/stellungnahmen/, Zugriff am 21.12.2023.
